# Cross-sectional associations between healthy and unhealthy plant-based diets and metabolic syndrome in three distinct French populations: a meta-analysis

**DOI:** 10.1017/S0007114525000376

**Published:** 2025-04-14

**Authors:** Clémentine Prioux, Sandra Wagner, Léopold K. Fézeu, Valérie Deschamps, Charlotte Verdot, Julia Baudry, Mathilde Touvier, Serge Hercberg, Julie-Anne Nazare, Axelle Hoge, João Pedro Ferreira, Patrick Rossignol, Nicolas Girerd, Sopio Tatulashvili, Emmanuelle Kesse-Guyot, Benjamin Allès

**Affiliations:** 1 Université Sorbonne Paris Nord and Université Paris Cité, Inserm, INRAE, CNAM, Nutritional Epidemiology Research Team (EREN), Center of Research in Epidemiology and StatisticS (CRESS), 74 rue Marcel Cachin, F-93017, Bobigny, France; 2 University of Lorraine, Inserm CIC 1433, Nancy CHRU, Inserm U1116, FCRIN, INI-CRCT, 4 rue du Morvan, 54500 Vandoeuvre-lès-Nancy, France; 3 Nutritional Epidemiology Surveillance Team (ESEN), Santé publique France, The French Public Health Agency, Bobigny, France; 4 Public Health Department, Hôpital Avicenne, Assistance Publique-Hôpitaux de Paris (AP-HP), Bobigny, France; 5 Centre de Recherche en Nutrition Humaine Rhône-Alpes, CarMeN lab, Univ-Lyon, Inserm, INRAe, Claude Bernard Lyon1 University, Centre Hospitalier Lyon Sud, Hospices Civils de Lyon, Pierre Bénite, France; 6 Department of Public Health, University of Liège, Liège, Belgium; 7 Cardiovascular R&D Centre-UnIC@RISE, Department of Physiology and Cardiothoracic Surgery, Faculty of Medicine of the University of Porto, Porto, Portugal; 8 Department of Internal Medicine, Heart Failure Clinic, Centro Hospitalar de Vila Nova de Gaia/Espinho, Vila Nova de Gaia, Portugal; 9 Medicine and Nephrology-Dialysis Departments, Princess Grace Hospital, and Monaco Private Hemodialysis Centre, Monaco, Monaco; 10 AP-HP, Avicenne Hospital, Paris 13 University, Sorbonne Paris Cité, Department of Endocrinology-Diabetology-Nutrition, CRNH-IdF, CINFO, 93000 Bobigny, France

**Keywords:** Plant-based diet, Cardiovascular risk factors, Dietary patterns, Multi-cohort study epidemiology, Meta-analysis

## Abstract

Prior studies have shown that plant-based diets are associated with lower cardiovascular risk. However, these diets encompass a large diversity of foods with contrasted nutritional quality that may differentially impact health. We aimed to investigate the pooled cross-sectional association between metabolic syndrome (MetS), its components and healthy and unhealthy plant-based diet indices (hPDI and uPDI), using data from two French cohorts and one representative study from the French population. This study included 16 358 participants from the NutriNet-Santé study, 1769 participants from the Esteban study and 1565 participants from the STANISLAS study who underwent a clinical visit. The MetS was defined according to the International Diabetes Federation definition. The associations between these plant-based diet indices and MetS were estimated by multivariable Poisson and logistic regression models, stratified by gender. Meta-analysis enabled the computation of a pooled prevalence ratio. A higher contribution of healthy plant foods (higher hPDI) was associated with a lower probability of having MetS (PR_men_: 0·85; 95 % CI: 0·75, 0·94, PR_women_: 0·72; 95 % CI: 0·67, 0·77), elevated waist circumferences and elevated blood pressure. In women, a higher hPDI was associated with a lower probability of having elevated triacylglyceride (TAG), low HDL-cholesterolaemia and hyperglycaemia; and a higher contribution of unhealthy plant foods was associated with a higher prevalence of MetS (PR_women_: 1·13; 95 % CI: 1·01, 1·26) and elevated TAG. A greater contribution of healthy plant floods was associated with protective effects on metabolic syndrome, especially in women. Gender differences should be further investigated in relation to the current sustainable nutrition transition.

Metabolic syndrome (MetS) is an increasingly prevalent issue, affecting between 12·5 % and 31·4 % worldwide and 31·5 % in Europe, depending on the study population characteristics and the diagnostic criteria^(1)^. MetS corresponds to concomitant metabolic abnormalities representing risk factors for cardiovascular diseases. It is characterised by five criteria: hypertriglyceridaemia, elevated blood pressure (BP), hyperglycaemia, abdominal obesity and dyslipidaemia^(2)^. MetS is highly associated with diabetes and cardiovascular diseases and can be used clinically in primary prevention to identify individuals at high risk of cardiovascular diseases and mortality^(3,4)^ or defined as a pre-morbid condition^(5)^.

Numerous studies have highlighted the importance of diet as a modifiable risk factor for MetS and, more generally, cardiovascular diseases^(6)^. More specifically, plant-based diets have gained significant popularity in recent years for their environmental and health benefits. Some studies and reviews have highlighted the benefits of diets rich in plant foods to reduce the risk of MetS^(6–8)^. Previous studies have shown that the nutritional quality of plant-based diets varies, which impacts their effectiveness in preventing cardiovascular disease^(9,10)^. One study reported that unhealthy plant-based diets can have detrimental effects on certain factors such as HDL and TAG. It could be hypothesised that some individuals transitioning to plant-based diets may substitute animal foods with ultra-processed plant foods rich in carbohydrates and sugars and even saturated fats, inducing them to both a reduced HDL-cholesterol and an increased TAG blood level^(11)^.

Several indices based on the contribution of plant foods to the diet compared with animal products have been proposed, and among them, plant-based diet indices (PDI) have been more frequently reported as associated with cardiovascular health^(8,12)^. The healthy plant-based diet indices (hPDI) reflect to which extent a diet is characterised by a higher consumption of healthy plant foods, such as vegetables, fruit and whole grains and a lower contribution of animal foods, whereas the unhealthy plant-based diet indices (uPDI) reflect to which extent a diet is characterised by a higher consumption of unhealthy plant foods such as refined grains and sugary drinks^(12)^. To our knowledge, very few studies have examined the link between these indices and MetS, especially in different gender groups. Most of these studies have been conducted in North America^(13,14)^ or Asia^(15–17)^ and fewer in Europe: one large epidemiological study in Spain^(18)^ and a smaller one in Denmark^(19)^, inducing a lack of results from this continent about the association between clinically measured risk factors of MetS and plant-based diets. Five of these studies reported that higher scores of hPDI were associated with a reduced risk of MetS^(13,16–19)^, and two others did not report any association^(14,15)^. In three of the studies, higher scores of uPDI were associated with an increased risk of MetS^(16,18,19)^, and three other studies did not report any association^(14,15,17)^. As the nutritional quality of plant-based diets varies across countries due to different cultural settings and associated food habits, it remains important to add new knowledge regarding cardiovascular risk factors and these diets in Europe, in large cohort studies.

Therefore, this study aims to investigate the pooled cross-sectional associations between MetS, its components and healthy and unhealthy plant-based diet indices (hPDI and uPDI) by gender, using data from two cohorts: NutriNet-Santé and STANISLAS and one representative survey: Esteban.

## Methods

### Study population and design

This study is based on two cohorts: NutriNet-Santé and STANISLAS and a national representative survey of the French population: Esteban.

- NutriNet-Santé:

The NutriNet-Santé cohort is a prospective online observational cohort launched in 2009. This study has been described in detail elsewhere^(20)^.

This cohort study was conducted in accordance with the Declaration of Helsinki and approved, and all procedures were approved by the Institutional Review Board of the French Institute for Health and Medical Research (IRB Inserm number 0000388FWA00005831) and the National Commission on Informatics and Liberty (CNIL numbers 908450 and 909216). All participants gave their informed consent electronically. The clinical trial number is NCT03335644.

Between 2011 and 2014, NutriNet-Santé participants were voluntarily asked to undergo a clinical examination and biological sampling in one of the local centres located throughout France. Informed consent has been retrieved for all participants. All procedures were submitted for approval to the ‘Consultation Committee for the Protection of Participants in Biomedical Research’ (C09-42 on 5 May 2010) and to the CNIL (No. 1460707) for the protection of participants in biomedical research.

- Esteban:

The Esteban survey is a representative cross-sectional study of French adults conducted between 2014 and 2016. The protocol for this survey has already been published^(21)^. The study has been registered with the French National Agency for Medicines and Health Products Safety) (No. 2012-A00456-34) and has been approved by the Advisory Committee for the Protection of Individuals in Biomedical Research.

The Esteban study used a three-stage probabilistic sampling design. In the first stage, a stratified sample of primary units (PU) was drawn at random. In the second stage, households in each PU were selected at random by telephone sampling. In the third stage, a single individual (adult or child) was drawn from among the eligible members of the household.

Stratification was based on two variables: region (eight geographical areas) and size of the urban residence unit (five strata: rural; < 20 000 inhabitants; 20 000–100 000 inhabitants; > 100 000 inhabitants, Paris). This complex survey design was taken into account in the estimation of the initial weighting applied to each person who participated in the first visit. This weighting corresponded to the number of eligible persons in the household, multiplied by the inverse of the probability of drawing from the household and by the inverse of the probability of drawing from the PU^(22)^.

- STANISLAS:

The STANISLAS cohort is a population-based study of 1006 families each comprised of at least two parents and two children (4295 participants) from the Lorraine region (Eastern France) recruited during 1993–1995 at the Center for Preventive Medicine. The participants were of French origin and free of acute or chronic disease. They participated at three follow-up visits, each every 5 to 10 years. From 2011 to 2016, 1705 participants underwent their fourth examination. The STANISLAS study has been described in detail elsewhere^(23)^. The present study focuses only on the fourth visit where a FFQ was administered.

The research protocols were all approved by the local Ethics Committee (Comité de Protection des Personnes Est III—Nancy—France) and all study participants gave written informed consent.

#### Clinical and biological data assessment and harmonisation

- NutriNet-Santé:

During the clinical examination, trained staff measured systolic and diastolic BP three times at 1-minute intervals in the seated position after lying down for 5 min using a validated automatic device (HEM-7015IT; OMRON, Rosny-sous-Bois, 130 France). For the analyses, mean values were calculated according to the three catches.

Anthropometric data were also collected during this examination by trained staff with standardised procedures. Height and weight were measured once using a wall cloth and an electronic scale (BC-418MA; TANITA, Tokyo, Japan) respectively. The BMI (kg/m^2^) was calculated. Waist circumference was measured by taking the circumference halfway between the lower ribs and the iliac crests.

During the clinical examination, blood samples were taken after a fasting period of at least 6 h and centralised for analysis in a single laboratory (IRSA, Tours, France). Measurements included total serum cholesterol (cholesterol oxidase C8000, Abbott), HDL-cholesterol (direct accelerator C8000, Abbott), serum TAG (glycerol kinase C8000, Abbott) and fasting blood glucose (hexokinase on C 8000 automat, Abbott, Suresnes, France). LDL-cholesterol was calculated using the Friedwald formula^(24)^.

Following the clinical assessment data regarding drug intakes were collected.

- Esteban:

Biological samples and measurements were taken during the clinical examination. BP was measured according to the method used in the European Health Examination Survey protocol^(25)^. BP was measured using a BP monitor (Omron 705-IT). Three measurements were taken 1 min apart, 30 min after the blood sample was taken and after 5 min of rest with no change in position.

Height and weight were measured once using a portable measuring (Leicester Tanita HR 001) rod and a scale (SECA 803 Clara), respectively. The BMI (kg/m^2^) was calculated.

Waist circumferences were measured, using a flexible tape measure placed midway between the last rib and the iliac crest in a horizontal plane. The measurement, in cm, was read at the end of a normal exhalation^(21)^.

During the clinical examination, a fasting blood sample and urine were taken from all participants. A lipid profile (total cholesterol, HDL, calculated LDL, TAG), blood sugar levels and a complete blood count were carried out.

- STANISLAS:

Anthropometric measurements, such as weight, height and waist circumference were performed during clinical examination. The BMI (kg/m^2^) was calculated. Office BP was also measured. After 10 min of rest in the supine position, systolic and diastolic BP were measured with an automatic device (Dinamap Pro 400, CRITIKON). Office BP was measured three times at 1-minute intervals and the mean of the three measures was considered. Blood samples were collected during the clinical examination and serum concentrations of the many biomarkers were measured including fasting glucose, HDL, calculated LDL-cholesterol and TAG^(23)^.

In the three studies, data regarding drug intakes were collected during or following the clinical examination.

Definition of MetS:

MetS was defined in the three studies according to the International Diabetes Federation criteria^(2)^. MetS is attributed to individuals having three or more of the five following criteria:

- elevated waist circumference (waist circumference ≥ 94 cm for men and ≥ 80 cm for women),
- elevated BP (SBP/DBP ≥ 130/85 mmHg or antihypertensive drug treatment),
- hypertriglyceridaemia (≥ 150 mg/dl or fibrate drug treatment),
- low HDL (< 40 mg/dl for men or < 50 mg/dl for women),
- hyperglycaemia (fasting glycemia > 100 mg/dl or antidiabetic drug treatment).

Clinical and biological data harmonisation enabled us to compute the components of MetS in each sample similarly.

#### Dietary data collection

- NutriNet-Santé:

At baseline and every 6 months thereafter, dietary data were collected using 24-hour dietary recalls, randomly distributed over 2 weeks comprising two weekdays and one weekend day^(26)^. Participants who completed at least three 24-hour recordings at inclusion were included in this study. The analyses were performed on the recordings collected when the participants were included in the study. Food Propensity Questionnaire was used to gather information on the frequency of consumption of occasionally consumed foods and drinks over the 12 months preceding the study (e.g. for better estimation of fish intake)^(27)^.

The participants reported all the foods they consumed throughout the day, which they chose from a list of approximately 3500 items of foods usually consumed in the French diet. Portion sizes were then estimated using purchase unit, household unit and photographs, derived from a previously validated picture booklet^(28)^. Daily intakes for energy, macro and micronutrients were estimated using the published NutriNet-Santé food composition table^(29)^, weighted according to the day (week or weekend). Dietary underreporters were identified by the method proposed by Black^(30)^. These web-based dietary records have been validated in several studies against traditional dietitians’ interviews^(31)^ and against biomarkers of nutritional status^(32,33)^. Dietary data were collected on mean 2 years and 1 month before the health examination (sd = 1 year and 2 months).

- Esteban:

Dietary data from the 24-hour recall method was collected by telephone with a dietitian or by the Internet. In this study, we included participants with at least two 24-hour recalls. The 24-hour recalls were randomly selected (2 weekdays and one weekend day), and the participants were not informed in advance so that they could not modify their dietary habits. In addition to the 24-hour recalls, a Food Propensity Questionnaire was used to gather information on the frequency of consumption of occasionally consumed foods and drinks over the 12 months preceding the study (e.g. for better estimation of fish intake)^(27)^.

- STANISLAS:

Dietary intake was assessed using a validated FFQ^(34)^. Over the past 3 months, participants reported their frequency of consumption and portion sizes for 133 foods and beverages. Frequency of consumption was recorded at six levels, ranging from ‘never or rarely’ to ‘2 or more times a day’. Portion sizes for each food or drink were estimated using standard portions and food models. Daily nutrient intakes were calculated in grams per day by multiplying the frequency of consumption of each item by the nutrient content of the selected portions. The nutritional data used was extracted from the French food composition database compiled by the Centre de Données sur la Qualité des Aliments (Ciqual).

#### Meat, plant-based foods and used indicators harmonisation and computation healthy plant-diet index and unhealthy plant-diet index

For all studies, foods and drinks were classified into eighteen food groups based on nutrients and culinary similarities, developed by Satija *et al.* ^(12)^, and were adapted for the three databases to better match with French consumption habits^(35)^ (online Supplementary 3). To compute dietary indices using the most similar methods as possible between the three sets of data, a first step of harmonisation work was carried out for the STANISLAS study. NutriNet-Santé and Esteban data sets were very similar in terms of food groups and dietary variables, whereas STANISLAS study used a different nutritional survey tool to estimate dietary intakes. Thus, for STANISLAS, some dishes such as sauerkraut and cassoulet evaluated by FFQ were converted into food groups, based on their composition estimated from generic recipes obtained from NutriNet-Santé and Esteban studies.

The hPDI and the uPDI were developed by the method of Sajita *et al.* ^(10,12)^ to reflect, respectively, the consumption of ‘healthy’ plant foods (hPDI) known to be associated with a lower risk for certain diseases and the consumption of ‘unhealthy’ plant foods (uPDI) known to be associated with a higher risk for certain diseases^(12)^, respectively. The computation methods of these indices were applied similarly in the three dietary databases following previous work from the NutriNet-Santé study^(35)^. The mean daily intakes for each participant were compared with the quintiles of consumptions of the eighteen food groups, of each study sample, following a reverse scoring system for healthy or unhealthy plant foods and animal foods. A higher score on all indices reflects a lower dietary intake of animal products. Healthy and unhealthy PDI range from 18 to 90. A higher hPDI means that the diet favours healthy plant foods over unhealthy plant foods, and vice versa for an uPDI. Further details about the methodology of these dietary indices adapted for the NutriNet-Santé study were previously published (online Supplementary 2)^(35)^.

- Animal/plant-based protein intake indices

Two other indicators, which were not taken into account for PDI computation, were used to assess the contribution of plant foods to the diet, in line with a previous study^(36)^. The first is the percentage of non-alcoholic energy intake provided by plant proteins calculated as

Portion of plant proteins (g)/ Alcohol-free energy intake (kcal) 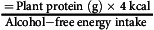 × 100

The second is the animal/plant proteins ratio, calculated as

Animal/plant proteins ratio = 
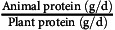


#### Covariates – data harmonisation

Data were collected using self-reported questionnaires for NutriNet-Santé and STANISLAS studies.

For Esteban study, data were mainly collected using questionnaires completed face-to-face by an interviewer visiting participants’ homes, and using self-administered questionnaires on paper or via Internet, depending on the choice made by the participants.

For the three studies, data collected included information on socio-demographic and socio-economic factors and lifestyle, such as gender, age, education (highest diploma obtained), household composition, socio-professional category and smoking habits. Place of residence was collected only for NutriNet-Santé and Esteban studies. Net monthly household income was assessed for all three studies, with categories differing between the three studies (Monthly household income categories: NutriNet-Santé: < 1430 €/ 1430–2000 €/ 2000–2700 €/ > 2700 €/ Refused to declare, STANISLAS: < 1499 €/ 1500–2249 €/ 2000–2700 €/ > 2700 €/ Refused to declare, Esteban: < 1300 €/ 1300–1900 €/ 1900–2500 €/> 2500 €).

Physical activity was assessed using the International Physical Activity Questionnaire^(37)^ for NutriNet-Santé and STANISLAS studies and by the recent Physical Activity Questionnaire^(38)^ for Esteban study.

A family history of myocardial infarction or sudden cardiac death before the age of 55 in the father and/or brother and/or son and before the age of 65 in the mother and/or sister and/or daughter was collected for Esteban study and a family history of infarction in the father, mother, brother and sister for NutriNet-Santé and STANISLAS studies.

In all three studies, participants were asked whether they were on a diet at the time the dietary data were assessed. In NutriNet-Santé and STANISLAS studies, a participant was considered to be on a diet for medical reasons or weight management (lose weight or keep it off or stay in shape). In Esteban study, a participant was considered to be on a diet for medical reasons/allergies/intolerances or weight management (to lose weight or keep it off or to gain weight/stay fit or out of conviction/other).

When the percentage of missing data was less than 2 %, we reclassified the missing data in the most represented category. Otherwise, a missing data category has been created.

### Statistical analysis

First, for the three studies: socio-demographic, anthropometric, lifestyle and physical activity characteristics; dietary data, indicators, scores; MetS and its components were described. Dietary data were adjusted for age and total energy intake.

Second, to evaluate the association between the scores (hPDI, or uPDI), modelled as a continuous (per ten unit increase), and MetS or its components:

- when the occurrence of the binary-dependent variable (MetS or syndrome component) was less than 10 %, we used logistic regression.
- when the prevalence of the dependent variable exceeds 10 %, OR derived from standard logistic regressions are not deemed suitable proxies for relative risks. Therefore, we used Poisson regression with a robust error variance, an alternative method recommended by Zou *et al.* ^(39)^.

We estimated prevalence ratios (PR) or OR and 95 % CI and *P*-values. For the three studies, the models were stratified by gender. For NutriNet-Santé and Esteban studies, models are adjusted for age, height, education level, household composition, place of residence, net monthly household income, socio-professional category, physical activity, smoking status, energy intake without alcohol (kJ/d), alcohol consumption (g/d), family history and personal-specific restrictive diet followed. The same adjustment factors were used for the STANISLAS study, except for place of residence and socio-professional category, in line with previous work based on the STANISLAS cohort^(40)^. All the adjustment factors were selected based on the literature review.

Finally, we computed a pooled PR (overall PR) using random-effects or fixed-effects meta-analysis of PR or OR from each study. Statistical heterogeneity was assessed with the Cochran *Q*-test (*P* < 0·10) and *I* ^2^ statistic. A random-effects model was employed if the heterogeneity *I*² value exceeded 50 %; otherwise, a fixed-effects model was chosen.

All tests were two-sided, and *P* < 0·05 was considered statistically significant. Statistical analyses were performed with SAS (version 9.4, SAS Institute, Inc.) and R studio (R version 4.2.2).

## Results

### Characteristics of participants

General characteristics of the three studies are shown in Table 1, and the selection for the study samples is presented in flowchart in supplementary data (online Supplementary 1A, B, C).

- NutriNet-Santé (2009–2014):

**Table 1.** Description of socio-demographic, anthropometric and lifestyle characteristics of the three studies, NutriNet-Santé (*n* 16 358), Esteban (*n* 1769) and STANISLAS (*n* 1565) studies (Numbers and percentages; mean values and standard deviations)

	NutriNet-Santé (*n* 16 358)		Esteban (*n* 1769)		STANISLAS (*n* 1565)	
	*n*	%	*n*	%	*n*	%
Gender (%)						
Male	4623	28·3	849	48·0	762	48·7
Women	11 735	71·7	920	52·0	803	51·3
Age (years) (%)						
18–30	1614	9·9	248	14·0	144	9·2
30–50	4935	30·2	660	37·3	489	31·3
50–65	7548	46·1	616	34·8	792	50·6
≥ 65	2261	13·8	245	13·9	140	8·9
Age (year)^*^						
Mean	50·9		47·6		49·1	
sd	13·6		14·5		14·0	
Monthly household income categories (%)^†^						
Very low	1129	6·9	218	12·3	197	12·6
Low	1676	10·2	253	14·3	298	19·0
Intermediate	2404	14·7	320	18·1	318	20·3
High	9741	59·6	876	49·5	700	44·7
Refused to declare	1408	8·6	102	5·8	52	3·3
Socio-professional category^‡^ (%)						
Unemployed	1631	10·0	70	4·0	163	10·4
Self-employed, farmer, employee, manual worker	2241	13·7	686	38·8	490	31·3
Intermediate profession	2509	15·3	387	21·9	86	5·5
Managerial staff, intellectual profession	3672	22·4	184	10·4	575	36·7
Students or retired people	6305	38·5	442	25·0	251	16·0
Educational level (%)						
None or primary	516	3·2	152	8·6	85	5·4
Secondary	5250	32·1	1058	59·8	572	36·6
Higher education	10 592	64·7	558	31·6	908	58·0
Household composition (%)						
Alone without children	3394	20·8	325	18·4	196	12·5
Alone with at least one child	905	5·5	186	10·5	53	3·4
Two adults living as a couple without children	6984	42·7	577	32·6	793	50·7
Two adults living as a couple with at least one child	4619	28·2	653	36·9	459	29·3
Two or more adults without children	456	2·8	28	1·6	64	4·01
Size of the urban residence unit^‡^ (%)						
Rural	3153	19·3	528	29·6	NA	NA
< 20 000 inhabitants	2423	14·8	310	17·5	NA	NA
20 000–200 000 inhabitants or 10 000–100 000 inhabitants	2590	16·8	256	14·5	NA	NA
> 200 000 or > 100 000 inhabitants	8059	49·3	676	38·2	NA	NA
Physical activity^‡^ (%)						
High physical activity	5646	34·5	669	37·8	470	30·0
Moderate physical activity	6202	37·9	915	51·7	348	22·2
Low physical activity	2760	16·9	185	10·5	474	30·3
Missing data	1750	10·7	0	0	273	17·4
Smoking status^‡^ (%)						
Smoker	1789	10·9	409	23·1	499	31·9
Former smoker	6540	40·0	454	25·7	320	20·4
Never smoked	8029	49·1	906	51·2	746	47·7
Family history of myocardial infarction sudden cardiac death before the age of 55^§^ (%)						
No or don’t know	12 502	76·4	1591	89·9	1310	83·7
Yes	3856	23·6	178	10·1	255	16·3
Diet currently followed^||^ (%)						
Yes	2944	18·0	388	21·9	146	9·3
No	13 414	82·0	1381	78·1	1419	90·7
BMI^*^,^¶^ (kg/m^2^)						
Mean	24·33		25·9		26·0	
sd	4·34		5·0		4·8	
BMI categories (%)						
< 25	10 443	63·8	890	50·3	763	48·7
25–30	4343	26·6	571	32·3	529	33·8
30–35	1117	6·8	225	12·7	199	12·7
35–40	326	2·0	56	3·2	49	3·1
≥ 40	129	0·8	27	1·51	25	1·6

* Mean and sd.

† Monthly household income categories: NutriNet-Santé: < 1430 €/1430–2000 €/2000–2700 €/ > 2700 €/ Refused to declare, STANISLAS: < 1499 €/ 1500–2249 €/2000–2700 €/ > 2700 €/ Refused to declare and Esteban: < 1300 €/ 1300–1900 €/ 1900–2500 €/ > 2500 €. The median standard of living for people living in a household in mainland France is €1692 per month^(41)^.

‡ In NutriNet-Santé-Santé study, the socio-professional category missing data (*n* 81, 0·5 %) are reclassified in the most represented category.

In NutriNet-Santé-Santé study, the size of the urban residence unit missing data (*n* 133, 0·8 %) are reclassified in the most represented category.

In Esteban study, the physical activity missing data (*n* 22, 1·2 %) are reclassified in the most represented category.

In NutriNet-Santé-Santé study, the smoking status missing data (*n* 3, 0·02 %) are reclassified in the most represented category.

In NutriNet-Santé-Santé study, the size of the urban residence unit missing data (*n* 3, 0·02 %) is reclassified in the most represented category.

In Esteban study, the size of the urban residence unit missing data (*n* 8, 0·45 %) is reclassified in the most represented category.

§ Family history of myocardial infarction in the father/mother and brother/sister or sudden cardiac death before the age of 55 in the father/mother and/or brother/sister and/or son/daughter).

|| In NutriNet-Santé-Santé and STANISLAS studies, a participant was considered to be on a diet for medical reasons or weight management (lose weight or keep it off or stay in shape). In the ESTEBAN study, a participant was considered to be on a diet for medical reasons/allergies/intolerances or weight management (to lose weight or keep it off or to gain weight/stay fit or out of conviction/other.

Of the 19 609 participants who participated in the clinical examination, 19 507 had valid socio-demographic and biological data at inclusion and 16 358 had valid dietary data, which is our final sample (28·3 % men and 71·7% women). The mean age was 50·9 (13·6) years. A total of 59·6% declared a high monthly household income (> 2700€, for your information: the median income per consumption unit in mainland France in 2009 was €1692 per month^(41)^). Among the participants, 22·4% were managers or in the intellectual profession, and 38·5% were students or retired people. A total of 49·3% lived in a city with more than 200 000 inhabitants. Among participants, 37·9% stated a high physical activity and 49·1% never smoked. A total of 23·6% had a family history of myocardial infarction. Among the participants, 18% followed a specific restrictive diet at the time dietary data was assessed. A total of 63·8% had a BMI < 25 kg/m^2^ and 26·6% had a BMI between 25 and 30 kg/m^2^.

- Esteban (2014–2016):

Of the 2496 participants with complete socio-demographic data, 1828 had valid health and biological data and 1769 had valid dietary data, constituting the final study sample, with 48 % men and 52% women. The mean age was 47·6 (14·4) years. A total of 49·5% declared a high monthly household income. Among the participants, 10·4% were managers or in the intellectual profession and 25% were students or retired people. A total of 38·2% lived in a city with more than 100 000 inhabitants. Among participants, 37·8% stated a high physical activity and 51·2% never smoked. A total of 10·1% had a family history of myocardial infarction or sudden cardiac death. Among the participants, 21·9% followed a diet at the time of dietary data was assessed. A total of 50·3% had a BMI < 25 kg/m^2^ and 32·3% had a BMI between 25 and 30 kg/m^2^.

- STANISLAS (2011–2016):

Of the 1632 participants with complete socio-demographic and dietary data, 1565 had valid health and biological data, constituting the final study sample (48·7 % men and 51·3% women). The mean age was 49·1 (14·0) years. A total of 44·7% declared a high monthly household income of more than 3000 euros. Among the participants, 22·5% were managers or in the intellectual profession and 38% were students or retired people. A total of 22·2% stated moderate physical activity and 47·7% never smoked. Among the participants, 16·3% had a family history of myocardial infarction. A total of 9·3% followed a diet at the time of dietary data was assessed. A total of 48·7% had a BMI < 25 kg/m^2^ and 33·8% had a BMI between 25 and 30 kg/m^2^.

### Prevalence of metabolic syndrome and its components

The prevalence of MetS and its components for the three studies are shown in Table 2.

**Table 2.** Description of the metabolic syndrome according to the International Diabetes Federation criteria and its components in the three studies, NutriNet-Santé (*n* 16 358), Esteban (*n* 1769) and STANISLAS (*n* 1565) studies (Numbers and percentages)

	NutriNet-Santé						Esteban						STANISLAS					
	All (*n* 16 358)		Women (*n* 11 735)		Male (*n* 4623)		All (*n* 1769)		Women (*n* 920)		Male (*n* 849)		All (*n* 1565)		Women (*n* 803)		Male (*n* 762)	
	*n*	%	*n*	%	*n*	%	*n*	%	*n*	%	*n*	%	*n*	%	*n*	%	*n*	%
Metabolic syndrome																		
No	14 215	86·9	10 473	89·2	3742	80·9	1454	82·2	789	85·8	665	78·3	1185	75·7	652	81·2	533	69·9
Yes	2143	13·1	1262	10·8	881	19·1	259	17·8	131	14·2	184	21·7	380	24·3	151	18·8	229	30·1
Elevated waist circumference																		
No	9581	58·6	6540	55·7	3041	65·8	901	50·9	430	46·8	471	55·4	690	44·1	333	41·5	357	46·8
Yes	6777	41·4	5195	44·3	1582	34·2	868	49·1	490	53·2	378	44·6	875	55·9	470	58·5	405	53·2
Elevated triglyceridemia																		
No	14 616	89·4	10 818	92·2	3798	82·1	1456	82·3	803	87·3	653	76·9	1132	72·3	634	78·9	498	65·3
Yes	1742	10·6	917	7·8	825	17·9	313	17·7	117	12·7	196	23·1	433	27·7	169	21·1	264	34·7
Elevated blood pressure																		
No	9134	55·8	7493	63·8	1641	35·5	1212	68·5	686	74·6	525	61·9	862	55·1	537	66·9	325	42·6
Yes	7224	44·2	4242	36·2	2982	64·5	557	31·5	234	25·4	324	38·1	703	44·9	266	33·1	437	57·3
Hyperglycaemia																		
No	14 150	86·5	10 518	89·6	3632	78·6	1660	93·8	888	96·5	772	90·9	1308	83·6	715	89·0	593	77·8
Yes	2208	13·5	1217	10·4	991	21·4	109	6·2	32	3·5	77	9·1	257	16·4	88	11·0	169	22·2
Low HDL																		
No	14 784	90·4	10 494	89·4	4290	92·8	1249	70·6	686	74·6	563	66·3	1315	84·0	654	81·4	661	86·7
Yes	1574	9·6	1241	10·6	333	7·2	520	29·4	234	25·4	286	33·7	250	16·0	149	18·6	101	13·3

The prevalence of MetS was the highest in the STANISLAS study (24·3 %) compared with Esteban (17·8 %) and NutriNet-Santé (13·1 %) studies.

The participants of the STANISLAS study also had higher waist circumference (55·9 %), hypertriglyceridaemia (27·7 %), elevated BP (44·9 %) and hyperglycaemia (16·4 %) compared with Esteban and NutriNet-Santé studies.

The prevalence of elevated waist circumference (41·4 %) and hypertriglyceridaemia (10·6 %) were the lowest in the NutriNet-Santé study compared with Esteban and STANISLAS studies.

Esteban study had the highest prevalence of low HDL (29·4 %) compared with STANISLAS (16·0 %) and NutriNet-Santé (9·6 %) studies, and the lowest prevalence of elevated BP and hyperglycaemia.

### Dietary data, indicators and scores

Description of the food groups consumption by the study is presented in Table 3. Indicators and scores of the three studies are presented in Table 4.

**Table 3.** Description of the dietary data of the three studies, NutriNet-Santé (*n* 16 358), Esteban (*n* 1769) and STANISLAS (*n* 1565) studies (Mean values with their standard errors of the means)

	NutriNet-Santé (*n* 16 358)				Esteban (*n* 1769)				STANISLAS (*n* 1565)					
	Women (*n* 11 735)		Male (*n* 4623)		Women (*n* 920)		Male (*n* 849)		Women (*n* 803)			Male (*n* 762)		
	m	sem	m	sem	m	sem	m	sem	m		sem	m	sem	
Healthy plant foods														
Wholegrain products (g/d)	40·40	0·50	41·80	0·90	37·90	1·90	31·80	2·10	21·20		1·90	20·80	1·96	
Fruit (g/d)	223·20	1·50	212·20	2·50	190·80	5·10	164·40	5·70	281·11		9·37	210·82	9·63	
Vegetables (g/d)	285·60	1·50	265·40	2·50	245·00	4·70	224·90	5·30	353·51		7·09	272·40	7·28	
Nuts (g/d)	5·60	0·10	3·20	0·20	2·50	0·20	1·20	0·20	3·28		0·26	2·20	0·26	
Legumes (g/d)	11·80	0·30	13·10	0·40	10·10	0·90	13·20	1·00	18·25		0·71	20·24	0·73	
Vegetable oils (g/d)	9·10	0·10	7·70	0·10	8·00	0·30	7·80	0·30	18·16		0·52	12·30	0·53	
Tea and coffee (ml/d)	515·50	3·40	349·80	5·70	458·40	10·50	350·50	11·80	540·0		13·32	422·55	13·69	
Total healthy plant foods	1091·3	4·60	893·2	7·60	952·7	14·0	793·8	15·80	1235·49		19·77	961·30	20·31	
Unhealthy plant foods														
Refined grains (g/d)	136·30	0·80	168·40	1·30	142·40	2·70	176·90	3·00	199·08		3·86	231·99	3·97	
Potatoes (g/d)	41·50	0·50	44·90	0·80	47·20	1·80	55·70	2·00	78·09		2·29	80·51	2·35	
Sugar-sweetened beverages (g/d)	82·90	1·10	96·90	1·90	92·50	4·90	99·40	5·60	108·04		6·33	123·15	6·51	
Sweets and desserts (g/d)	110·90	0·60	90·90	1·00	123·50	2·20	98·40	2·50	84·34		1·90	83·65	1·95	
Miscellaneous plant-based food (g/d)^*^	2·90	0·10	2·80	0·20	9·20	0·80	6·70	0·90	2·11		0·15	3·10	0·15	
Total unhealthy plant foods	374·4	1·40	403·90	2·40	414·8	5·70	437·1	6·40	471·66		7·27	522·40	7·47	
Animal foods														
Animal fat (g/d)	7·40	0·10	6·10	0·10	8·00	0·30	7·00	0·30	7·38		0·33	6·25	0·34	
Dairy products (g/d)^†^	236·90	1·50	224·50	2·60	218·70	4·80	198·60	5·40	300·74		7·75	259·04	7·96	
Egg (g/d)	14·10	0·20	13·40	0·30	12·80	0·70	12·30	0·70	14·78		0·56	14·89	0·58	
Seafood (g/d)	46·10	0·50	47·90	0·80	33·30	1·40	35·20	1·50	47·95		1·25	44·47	1·28	
Meat (g/d)^‡^	91·20	0·60	105·70	0·90	103·50	2·00	125·90	2·30	126·56		2·75	136·11	2·82	
Miscellaneous animal-based foods (g/d)^§^	27·0	0·40	28·0	0·70	54·20	2·80	61·30	3·20	57·39		1·70	72·21	1·75	
Total animal foods	425·60	1·70	425·5	2·80	430·5	5·50	440·3	6·10	554·80	8·06		532·98		8·28

* Values are means adjusted for age and total energy intake; sem: standard error of the mean.

† ‘Miscellaneous plant-based foods’ group includes plant-based sugary or salty snacks.

‡ ’‘Dairy’ products group includes butter, milk, cheese, yoghurts, cottage cheese, petits suisse and dairy product desserts. ‘Meat’ group includes meat, offal, processed meat, poultry, pork and poultry ham.

§ ‘Miscellaneous animal foods’ group includes all dressings, sauces and animal-based salty snacks and fast foods.

**Table 4.** Description of the indicators and scores of the three studies, NutriNet-Santé (*n* 16 358), Esteban (*n* 1769) and STANISLAS (*n* 1565) studies (Mean values and standard deviations)

	NutriNet-Santé (*n* 16 358)		Esteban (*n* 1769)		STANISLAS (*n* 1565)	
	m^*^	sd ^†^	m^*^	sd ^†^	m^*^	sd ^†^
Alcohol-free energy intake (kJ/d)	7941.2·0	2508.7	8036.6	2400.8	9585.1	3340.1
Alcohol consumption (g/d)	9·3	12·8	10·6	15·3	9·7	13·2
Contribution of plant foods to the diet						
Portion of plant proteins/alcohol-free energy intake (%)	5·7	1·5	5·0	1·1	5·4	1·3
Animal/plant proteins ratio (g/d)	2·3	1·4	2·6	1·1	2·4	1·2
Plant foods indices						
hPDI	55·5	7·6	53·6	7·1	52·82	8·5
uPDI	56·4	6·7	58·8	6·5	55·69	7·2

Range for hPDI in NutriNet-Santé: 30·0–84·0; uPDI: 32·0–80·0.

Range for hPDI in STANISLAS: 30·0–75·0; uPDI: 31·0–77·0.

Range for hPDI in Esteban: 28·0–82·0; uPDI: 38·0–76·0.

hPDI, healthy plant-based diet indices; uPDI, unhealthy plant-based diet indices.

* m: mean.

† sd: standard deviation.

Women in the NutriNet-Santé study had a fairly high consumption of wholegrain products and nuts; and men had a high consumption of wholegrain products, fruits, nuts and seafood.

Women in the STANISLAS study consumed high amounts of fruits, vegetables, legumes, vegetable oil, tea and coffee, refined grains, potatoes, sugar-sweetened beverages, dairy products, eggs, seafood, meat and miscellaneous animal-based foods; and men high amounts of vegetables, legumes, vegetable oil, tea and coffee, refined grains, potatoes, sugar-sweetened beverages, dairy products, eggs, meat and miscellaneous animal-based foods.

Women in the Esteban study consumed high amounts of sweets and desserts, miscellaneous plant-based food and animal fat; and men had high amounts of sweets and desserts, miscellaneous plant-based food and animal fat.

Even though the average values are close between the three studies, NutriNet-Santé represented the highest contribution of plant foods to the diet, with both the highest contribution of plant proteins and the lowest animal/plant proteins ratio.

### Association between the hPDI and uPDI scores and MetS and its components

**Figure 1.**
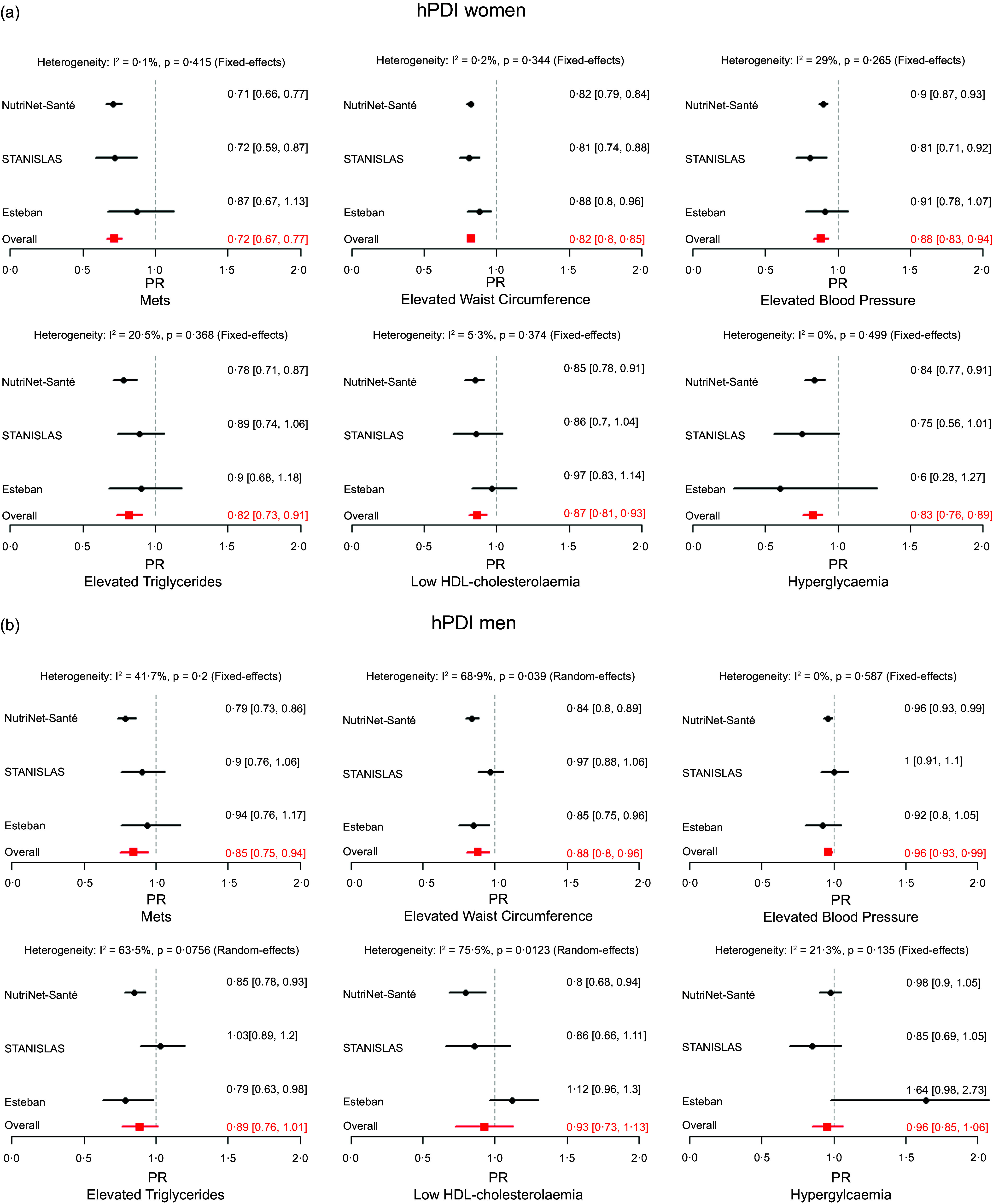
(a) Forest plot of studies (NutriNet-Santé-Santé (*n* 11 735), Esteban (*n* 920) and STANISLAS (*n* 803) studies) examining the association between MetS and its components and hPDI in continuous with 10-unit in women using random or fixed-effects meta-analysis. PR, prevalence ratios; MetS, metabolic syndrome; PDI, plant-based diet. For NutriNets-Santé and Esteban studies, the model was stratified by gender and adjusted for age, height, education level, household composition, place of residence, net monthly household income, socio-professional category, physical activity, smoking status, alcohol-free energy intake (kcal/d), alcohol consumption (g/d), family history and diet followed. For STANISLAS study, model stratified by gender and adjusted for age, height, education level, household composition, physical activity, smoking status, alcohol-free energy intake (kcal/d), alcohol consumption (g/d), family history and diet followed. (b) Forest plot of studies (NutriNet-Santé-Santé (*n* 4623), Esteban (*n* 849) and STANISLAS (*n* 762) studies) examining the association between MetS and its components and hPDI in continuous with 10-unit in men using random or fixed-effects meta-analysis. PR, prevalence ratios; MetS, metabolic syndrome; PDI, plant-based diet. For NutriNets-Santé and Esteban studies, the model was stratified by gender and adjusted for age, height, education level, household composition, place of residence, net monthly household income, socio-professional category, physical activity, smoking status, alcohol-free energy intake (kcal/d), alcohol consumption (g/d), family history and diet followed. For STANISLAS study, the model was stratified by gender and adjusted for age, height, education level, household composition, physical activity, smoking status, alcohol-free energy intake (kcal/d), alcohol consumption (g/d), family history and diet followed.

**Figure 2.**
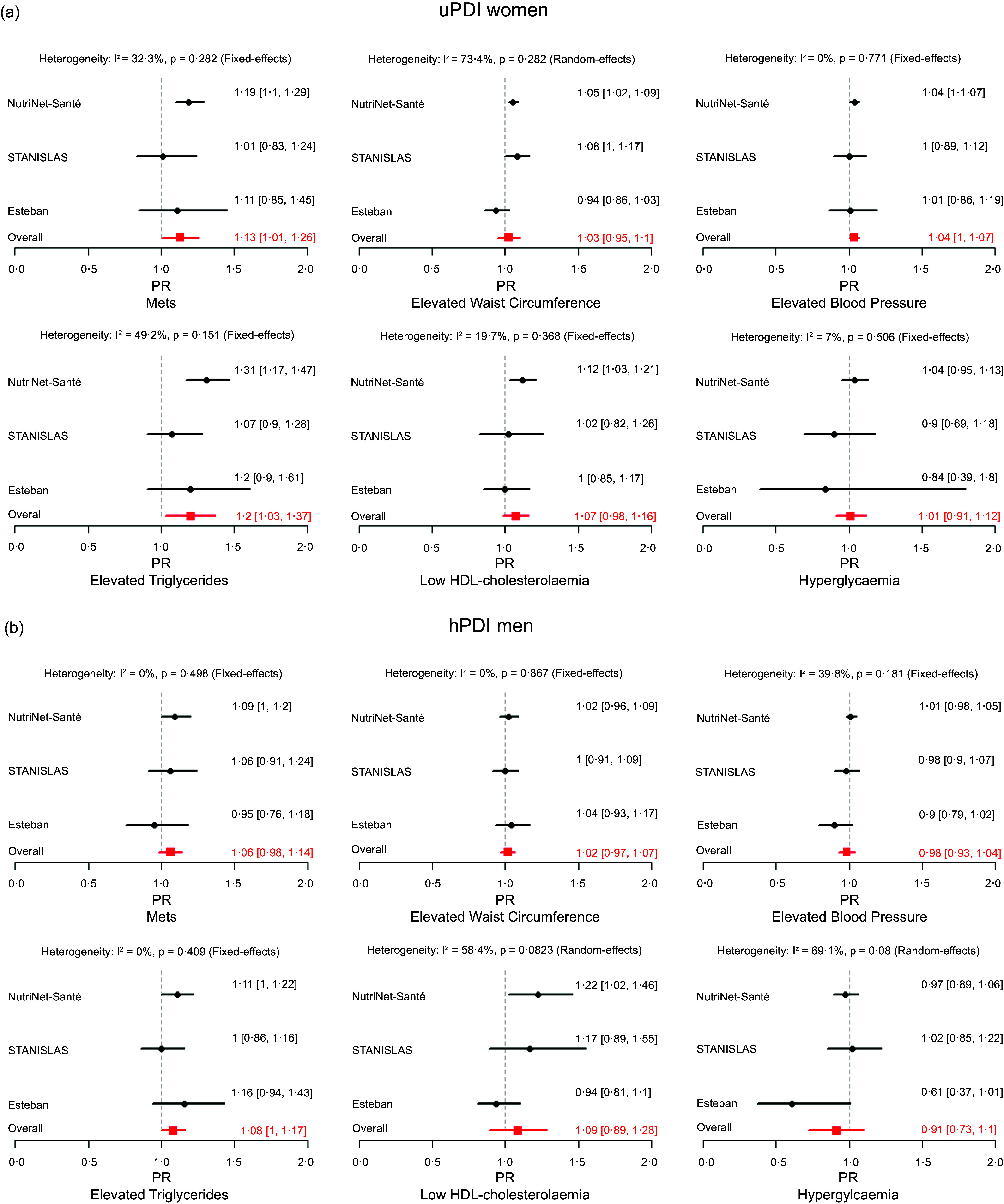
(a) Forest plot of studies (NutriNet-Santé-Santé (*n* 11 735), Esteban (*n* 920) and STANISLAS (*n* 803) studies) examining the association between MetS and its components and uPDI in continuous with 10-unit in women using random or fixed-effects meta-analysis. MetS, metabolic syndrome; PDI, plant-based diet; PR, prevalence ratios. For NutriNets-Santé and Esteban studies: model stratified by gender and adjusted for age, height, education level, household composition, place of residence, net monthly household income, socio-professional category, physical activity, smoking status, alcohol-free energy intake (kcal/d), alcohol consumption (g/d), family history and diet followed. For STANISLAS study, the model was stratified by gender and adjusted for age, height, education level, household composition, physical activity, smoking status, alcohol-free energy intake (kcal/d), alcohol consumption (g/d), family history and diet followed. (b) Forest plot of studies (NutriNet-Santé-Santé (*n* 4623), Esteban (*n* 849) and STANISLAS (*n* 762) studies) examining the association between MetS and its components and uPDI in continuous with 10-unit in men using random or fixed-effects meta-analysis. MetS, metabolic syndrome; PDI, plant-based diet; PR, prevalence ratios. For NutriNets-Santé and Esteban studies, the model was stratified by gender and adjusted for age, height, education level, household composition, place of residence, net monthly household income, socio-professional category, physical activity, smoking status, alcohol-free energy intake (kcal/d), alcohol consumption (g/d), family history and diet followed. For STANISLAS study, the model was stratified by gender and adjusted for age, height, education level, household composition, physical activity, smoking status, alcohol-free energy intake (kcal/d), alcohol consumption (g/d), family history and diet followed.

Multivariable PR or OR and 95 % CI, for each study, and from pooled meta-analyses, for MetS and its components according to the hPDI and uPDI scores in continuous with 10-unit increase are shown in Figure 1 and Figure 2.

After adjustments for potential confounding factors, in men and women, a higher contribution of healthy plant foods (higher hPDI mean scores) was associated with a lower prevalence of MetS (PR_men_: 0·85; 95 % CI: 0·75, 0·94; I^2^ = 41·7 %, PR_women_: 0·72; 95 % CI: 0·67, 0·77; I^2^ = 0·1 %), elevated waist circumferences (PR_men_: 0·85; 95 % CI: 0·75, 0·94; I^2^ = 68·9 %, PR_women_: 0·82; 95 % CI: 0·80, 0·85; I^2^ = 0·2 %) and elevated BP (PR_men_: 0·96; 95 % CI: 0·93, 0·99; I^2^ = , PR_women_: 0·88; 95 % CI: 0·83, 0·94; I^2^ = 29 %).

In women, a higher hPDI was associated with a lower probability of having elevated TAG (PR_women_: 0·82; 95 % CI: 0·73, 0·91, I^2^ = 20·5 %), low HDL-cholesterolemia (PR_women_: 0·87; 95 % CI: 0·81, 0·93, I^2^ = 5·3 %) and hyperglycaemia (PR_women_: 0·83; 95 % CI: 0·76, 0·89, I^2^ = 0 %).

In women, a higher contribution of unhealthy plant foods (higher uPDI mean scores) was associated with a higher prevalence of MetS (PR_women_: 1·13; 95 % CI: 1·01, 1·26, I^2^ = 32·3 %) and elevated TAG (PR_women_: 1·20; 95 % CI: 1·03, 1·37, I^2^ = 49·3 %).

## Discussion

Meta-analyses indicated that a higher contribution of healthy plant food was associated with a lower probability of having MetS, elevated waist circumferences and elevated BP, and only in women having elevated TAG, low HDL-cholesterolaemia and hyperglycaemia. We also observed in women that a higher contribution of unhealthy plant food was associated with a higher prevalence of having a MetS and elevated TAG.

Only two longitudinal studies investigated the association between MetS (and its components) and hPDI and uPDI. The first one is a Chinese study using data from the China Health and Nutrition Survey. The study included 10 013 participants with a median follow-up of 5 years. It reported that the highest quintile of hPDI had a 28 % risk of developing MetS and 20 % lower risk of developing abdominal obesity than those in the lowest quintile of hPDI. No statistically significant differences were found between hPDI and the other components of MetS^(17)^. The second one is a South Korean prospective cohort study including 5646 participants with a median follow-up of 8 years. This study did not highlight any association between hPDI with MetS and its components^(16)^.

Most cross-sectional studies investigating the associations between MetS (and its components) and plant-based diets reported results consistent with ours. A higher hPDI score was associated with a lower probability of having MetS in several studies, including the Danish MAX study^(19)^, the PREDIMED-Plus cohort (Spain)^(18)^ and the NHANES study (USA)^(13)^. Associations between higher hPDI and a reduced risk of elevated waist circumference were reported in the MAX study^(19)^, the NHANES ^(13)^ study and in cross-sectional study including participants of South Asian ancestry conducted in the USA^(14)^. Similarly, the PREDIMED-Plus cohort found a link between a healthy provegetarian score (similar to hPDI) and a lower BMI and waist-to-hip ratio^(18)^. The MAX study^(19)^ identified a protective association between hPDI and elevated BP. Two studies found that a higher hPDI was associated with a lower probability of presenting high LDL cholesterol or low HDL-cholesterol^(14,19)^. The South Asian study found that higher hPDI scores were linked to lower glycated haemoglobin level^(14)^.

To the best of our knowledge, only one cross-sectional study in South Korea did not find any association between hPDI and MetS^(15)^. Altogether, this confirms the external validity of our results, which add up new evidence that high intakes of healthy plant foods only, not all plant foods, may be protective against cardiovascular risk factors.

We found that in our study, the uPDI was associated with a higher prevalence of having a MetS and elevated TAG but only in women. The Chinese longitudinal study using data from the China Health and Nutrition Survey reported that those in the highest quintile of uPDI had a 36 % risk of developing incident abdominal obesity, compared with those in the lowest quintile of uPDI^(17)^. No association between uPDI with MetS and its other components was found in this study. In the South Korean longitudinal prospective cohort, it was observed that those in the highest quintile of uPDI had a 50 % higher risk of developing incident MetS compared with those in the lowest quintile of uPDI and greater adherence to uPDI was significantly associated with abdominal obesity, hypertriglyceridaemia, low HDL-C and elevated BP^(16)^.

The PREDIMED-Plus (Spain)^(18)^ and the cross-sectional Danish MAX^(19)^ studies found that higher uPDI scores were associated with a higher likelihood of MetS and low HDL-C levels. Similarly, the South Asian study highlighted that higher uPDI scores were associated with lower LDL cholesterol. The PREDIMED-Plus study^(18)^ identified positive associations between uPDI and plasma TAG, diastolic BP and plasma glucose levels. Similarly, the South Korean study found that a higher uPDI score was associated with higher odds of hypertriglyceridaemia in men and abdominal obesity, high fasting glucose and hypertriglyceridaemia in women^(15)^.

We hypothesise that cultures and related food habits vary between countries, possibly explaining discrepancies in results, especially between studies conducted in different continents. For example, the study which included participants of Korean adults did not report any association between healthy plant-based diets and MetS in contrast to our study (for the two cohorts)^(15)^. This study also contrasts with other longitudinal studies which reported that healthy plant-based diets were inversely associated with weight gain, incident obesity, hypertension and type 2 diabetes^(10 12 72 73 74)^. The authors hypothesised that this may be due to cultural differences between Western populations and Asian populations who already have a diet rich in plant foods where vegetables are often incorporated into all meals as side dishes. In turn, differences in dietary intake measured by hPDI may be less pronounced than in the Western population, limiting the ability to detect an inverse association between hPDI and MetS. In some countries, it is more important and necessary to promote the diversification of plant-based products, while in other countries or regions, the emphasis should initially be on reducing meat consumption.

The meta-analysis conducted in our study reported low levels of heterogeneity between the studies. When comparing the associations between the three cohorts, we observed more statistically significant associations in the NutriNet-Santé cohort. We also observed that the direction of these associations was the same in the three studies. It is noteworthy that NutriNet-Santé contains nine times more participants than the two other studies, representing the largest statistical power. This could explain why the associations are more significant in this study. Another hypothesis relies on the fact that different eating habits between countries and regions have an impact on the heterogeneity of the results. In France, for example, it is known that French people living in the Grand-Est region (where the STANISLAS study was carried out) consume more meat, particularly pork, than the national average^(42)^. It is also difficult to compare these two samples because the participants in the STANISLAS study have high intakes of both healthy plant and animal products, whereas the participants in the NutriNet-Santé study have high intakes of healthy plant-based products, but lower intakes of animal products. In addition, we also observed that NutriNet-Santé had the highest mean contribution of plant foods to the diet. This result could be explained by individual characteristics such as level of education and socio-professional category. For example, we know that people with a higher level of education eat more fruit and vegetables and, manual workers eat more meat and animal products^(43)^.

In our study, some associations between hPDI and uPDI and MetS or its components were only observed in women. Other studies have also highlighted this difference between genders. A previous study observed that the positive associations between uPDI and abdominal obesity, high fasting glucose and hypertriglyceridaemia were only observed in women^(15)^. Another study found that the association between uPDI and MetS was stronger in women (OR: 1·62, IC à 95 % 1·26–2·09, *P*-trend = 0·01) than in men (OR: 1·35, IC à 95 % 1·03–1·76, *P*-trend = 0·02). In the same study, hPDI had a significant protective effect in women, but no significant results were found in men^(17)^.

In our study, consumption of healthy plant foods was higher among women than men, while consumption of less healthy plant foods and animal products was lower among women. One possible explanation is that women are more likely to adopt healthy plant-based diets because they are more concerned about ethics and the environment^(44)^.

We also observed only in women an association between a higher consumption of less healthy plant foods and increased prevalence of MetS, and higher TAG only in women from the NutriNet-Santé cohort. There are only very few studies investigating the association between MetS, its components and plant-based diets by gender. A previous study reported an interaction between gender and age in their model and did observe a protective effect of being a woman^(13)^. This effect diminished in a group of women over 60 years^(13)^. This is in line with the existing literature, which shows that the prevalence of MetS increases with age, and this increase is more marked in women^(45)^. The mechanisms associated with this increased risk of cardiovascular disease after the age of 50 are not yet well explained in the literature. The hypothesis of the impact of menopause is often put forward^(46,47)^ but remains controversial^(48,49)^.

Our results strengthen the current public health nutritional guidelines about the beneficial effect of healthy plant foods on pathophysiological mechanisms of cardio-metabolic outcomes. A healthful plant-based dietary patterns is rich in fibres found in vegetables, legumes, wholegrain cereals, among others and nutrients that increase satiety with a low-calorie intake^(50)^, preventing increased waist circumference. Additionally, these compounds reduce cholesterol absorption, with a potential effect of reducing LDL cholesterol, and moderate postprandial insulin responses which will help to keep blood sugar levels stable^(51)^.

Some plant foods such as nuts, fruit, vegetables, spices and olive oil are particularly rich in antioxidants, particularly polyphenols, carotenoids and flavonoids, but also minerals involved in cardiovascular and circulation health such as potassium or Mg. Antioxidants can play several roles, such as protecting against oxidative stress, inhibiting platelet aggregation and reducing inflammation linked to visceral adiposity^(52,53)^. In addition, minerals in fruit and vegetables such as Potassium, for its beneficial effects on endothelial function and vascular homeostasis^(54,55)^ and Mg for its effects on carbohydrate metabolism, insulin sensitivity and anti-inflammatory, vasodilatory and anti-arrhythmic properties^(56,57)^ would prevent elevated BP but also hyperglycaemia.

Eating healthy plant foods could also have a beneficial impact on the intestinal microbiota^(8,58)^. Interactions between the microbiota and the human host can influence inflammation, nutrient metabolism, appetite regulation and the production of microbial metabolites, all important elements which were previously reported associated with the pathogenesis of MetS^(8,58)^.

Healthy plant-based diets have also been shown to improve blood lipid profiles as rich in monounsaturated fatty acids instead of saturated fatty acids from animal foods (e.g. red and processed meat, which are rich in SFA. This may induce an increase in HDL-cholesterol levels and a reduction of LDL cholesterol levels. These PUFA will also improve insulin sensitivity and prevent type 2 diabetes by modifying the fatty acid composition of the cell membrane and acting on the inflammatory response^(51)^.

A higher risk of having MetS when consuming a high amount of unhealthy plant foods like sweets, fries and white bread, may be explained by the pathophysiological mechanisms due to a diet rich in simple carbohydrates, saturated fats and salt contained in these foods^(9)^. This diet also often results in lower levels of micronutrients, antioxidants, dietary fibre and unsaturated fats, which are known to be protective against cardiovascular health outcomes, for example through diets such as the Mediterranean diet^(59)^. Additionally, higher levels of added sugars and a higher glycaemic load may be related to higher levels of inflammation (notably by IL-6, pro-inflammatory cytokines that have been associated with insulin resistance, type 2 diabetes^(60)^ and contribute to higher HDL levels^(57)^. It is noteworthy both in our study and in previous studies that hPDI was more frequently associated with MetS and its components than uPDI. A recent scoping review also reported that hPDI level was more frequently reported as associated with favourable outcomes whereas the uPDI was less frequently reported as associated with unfavourable outcomes for diabetes and cardiovascular diseases, consistent with our findings^(61)^. These results suggest that public health messages should focus on promoting a balanced diet containing a majority of healthy plant foods, such as fresh fruit and vegetables, whole bread and cereals, and limiting consumption of less healthy plant foods without necessarily excluding them from the diet.

### Strengths and limitations

The first limitation is the cross-sectional design of the study which limits our ability to establish causality and can lead to reverse causality.

Another limitation relies on the construction of the plant-based dietary indices (PDI): they do not consider plant-based meat analogues (e.g. soya burger patties, soya or almond milk, etc.). This category was not integrated into the PDI scores because of included foods which are highly heterogeneous in terms of nutritional quality and lack of knowledge regarding the impact on the health of these foods^(62,63)^. It is noteworthy that some of these plant foods are ultra-processed, a type of foods that has been associated with detrimental effects on health when consumed in excess^(64,65)^. A study has also reported that some of these products are high in nutrients that should be limited, such as salt and saturated fats^(66)^. It remains impossible with the current level of scientific evidence to categorise plant-based meat analogues within the healthy or unhealthy plant foods categories.

The studies have been performed in different time points which could have affected the results as dietary quality has shown declines currently compared with the past but these time points are very close (2009–2014 for NutriNet-Santé; 2014–2016 for Esteban and 2011–2016 for STANISLAS). It is possible that the nutritional quality of plant-based diets may have evolved and could be different from traditional diets. This may be due to the increasing availability of novel plant-based meat alternatives. However, a previous study carried out in the EPIC cohort reported that dietary quality and adherence to a healthy Mediterranean diet increased in most participants over time^(67)^. Thus, further studies could be conducted to assess whether changes in the nutritional quality of a plant-based diet over time may be associated with the risk of developing MetS. Although our analysis was based on the consensual definition of MetS, it did not take into account individual characteristics such as age, gender or smoking status^(68,69)^. However, adjustment for the individual characteristics may have reduced the impact of this limitation in our analyses.

We can also mention that different dietary data collection tools were used in the three studies. For example, dietary data in the STANISLAS study was based on an FFQ which better captures the consumption of rarely consumed foods, but may also lead to over-reporting the intake for specific food groups such as vegetables and legumes compared with the 24-hour recall method^(70)^. NutriNet-Santé and Esteban used a 24-h record which is nearly identical to the Esteban dietary survey was developed based on the NutriNet-Santé 24 h dietary record tool. This tool enables to register a wide variety of foods and limits the risk of overestimating the intake of some food groups such as fruits or vegetables but in case on a limited number of record days may miss some rarely consumed foods such as legumes, nuts or shellfish. However, we can note that the direction of the associations was similar between the three studies.

Another limitation relies on the relatively lower statistical power in STANISLAS and ESTEBAN compared with NutriNet-Santé to detect statistically significant associations.

A strength of our study is that we carried out a careful harmonisation. The advantages of using three studies are similar to those of multicentric studies^(71)^. Also, the low heterogeneity observed reinforces the external validity of our pooled estimators.

Additional strengths include the use of validated dietary assessment tools (FFQ and 24-hour recalls) and the quality of the information gathered by qualified professionals during the clinical examination guaranteeing the accuracy of our results.

This study is the first French study on this subject. It contributes to the existing literature by differentiating the effects of healthy and unhealthy plant diets on MetS and its components, analysing gender differences and drawing on data from a variety of studies with a very large study sample for pooled analyses.

### Conclusion

This study suggests that, among French adults, a greater adherence to healthy plant-based diets is associated with a lower probability of having a MetS. This protective effect was mostly observed with the components of MetS in women. These results are in line with other studies reporting that it is important to consider the nutritional quality of plant foods consumed in primary prevention for cardiovascular risk factors. Public health messages should focus on a diet with a high proportion of healthy plant-based foods, while limiting unhealthy plant foods, as they prevent cardiovascular risk factors such as MetS. Further longitudinal studies stratified by gender are required to confirm our results regarding the association of healthy plant-based diets with cardiovascular health outcomes and the potential protective effects against clinical damage to the heart and blood vessels.

## Supporting information

Prioux et al. supplementary material Prioux et al. supplementary material

## Supplementary material

For supplementary material/s referred to in this article, please visit https://doi.org/10.1017/S0007114525000376
