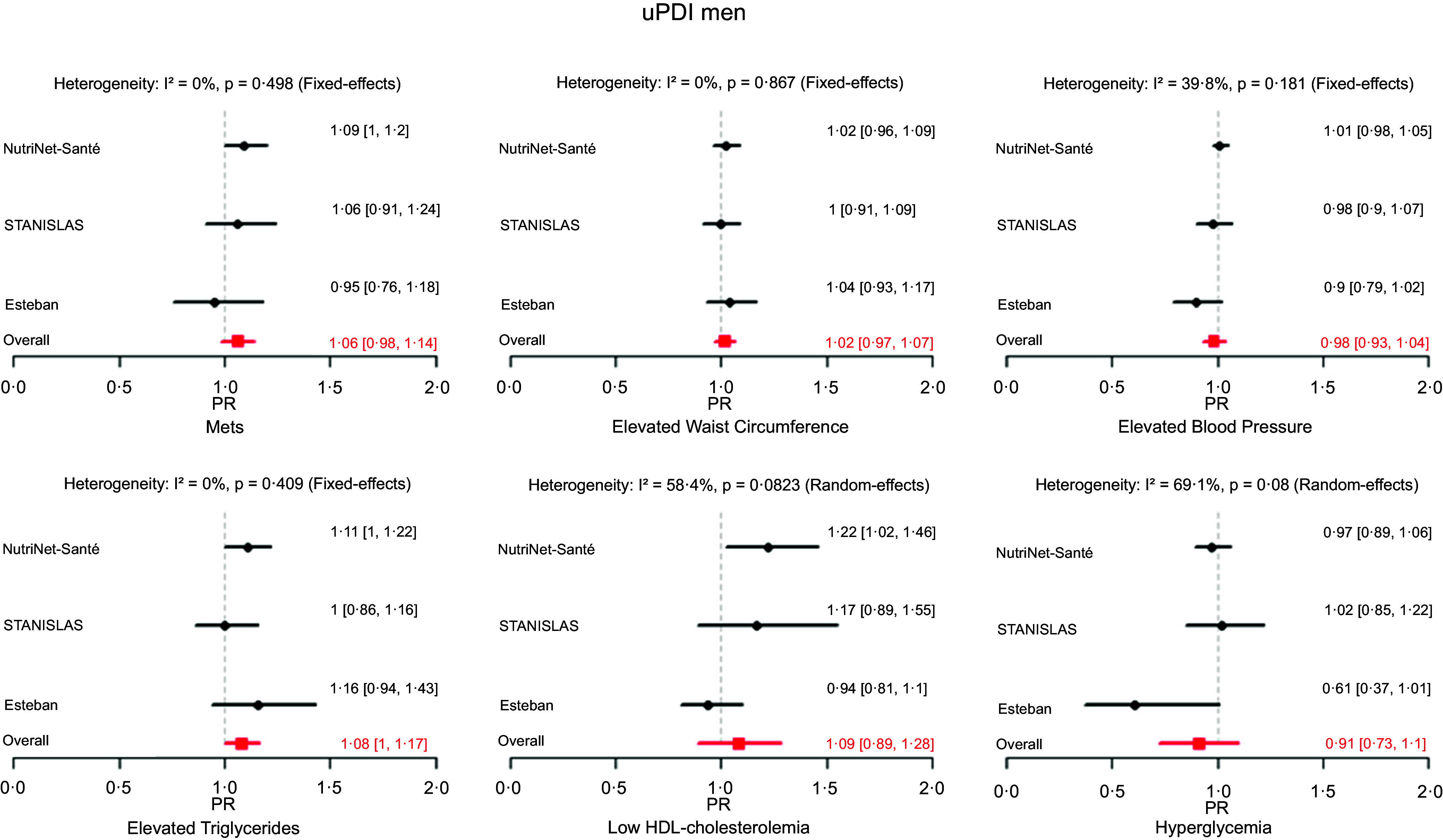# Cross-sectional associations between healthy and unhealthy plant-based diets and metabolic syndrome in three distinct French populations: a meta-analysis – ERRATUM

**DOI:** 10.1017/S0007114525103978

**Published:** 2025-08-14

**Authors:** Clémentine Prioux, Sandra Wagner, Léopold K. Fézeu, Valérie Deschamps, Charlotte Verdot, Julia Baudry, Mathilde Touvier, Serge Hercberg, Julie-Anne Nazare, Axelle Hoge, João Pedro Ferreira, Patrick Rossignol, Nicolas Girerd, Sopio Tatulashvili, Emmanuelle Kesse-Guyot, Benjamin Allès

The error concerns Figure 2 (a) second panel of graphs currently entitled “hPDI men”, this title is incorrect. The correct title is: “uPDI men” (not hPDI). This has been updated in the original publication. The correct version of the figure can be seen below:

**Figure:**