# Supplementary material for: Cross-sectional associations between healthy and unhealthy plant-based diets and metabolic syndrome in three distinct French populations: a meta-analysis
Source: Br J Nutr. 2025 Mar 3;133(7):949–65. doi: 10.1017/S0007114525000376 (PMC12198348; doi:10.1017/S0007114525000376)
Supplement: Prioux et al. supplementary material [file S0007114525000376sup001.docx]

SUPPLEMENTARY DATA

Supplemental 1: Flowchart of the three studies

Active individual with biobank data at inclusion

n= 19,507

Individuals with insufficient number of questionnaires n=1,481:
- exclusion of surveys with zero quantities
- exclusion of pregnant women at the time of filling
- exclusion of surveys with >10% of foods with quantity > maximum threshold and usual or lower consumption
- exclusion of surveys with quantities per group > maximum threshold

Participants with enough dietary questionnaires

n= 18,026

Participants with dietary, sociodemographic and health data completed n=16,358

Under-reporting and undetected participants

N=1,668

Participants who have data at inclusion
n= 19,609

Missing data at inclusion or unusable biobank data n=102

A: Flowchart of the NutriNet-Santé study

Participants who have sociodemographic data and health data
n= 1,828

Participants who have sociodemographic, heath and dietary data and health data
n= 1,769

Missing dietary data n=59

Participants with complete sociodemographic data
n= 2,496

Missing biological or health data n=668

B: Flowchart of the Esteban study

Participants who have biological and health data
n= 1,565

Participants with complete sociodemographic and dietary data
n= 1,632

Missing health or biological data n=67

C: Flowchart of the STANISLAS study

Supplemental 2: Food groups used to calculate PDI, hPDI and uPDI.

|  |  |  | **hPDI** | **uPDI** |
| --- | --- | --- | --- | --- |
| **Plant-based foods** | **Healthy** | Whole grains | Positive score  (1 to 5 based on consumption quintiles) | Reverse score  (5 to 1 based on consumption quintiles) |
|  |  | Fruits |  |  |
|  |  | Vegetables |  |  |
|  |  | Nuts |  |  |
|  |  | Legumes |  |  |
|  |  | Vegetable oil |  |  |
|  |  | Tea and coffee |  |  |
|  | **Unhealthy** | Refined grains | Reverse score  (5 to 1 based on consumption quintiles) | Positive score  (1 to 5 based on consumption quintiles) |
|  |  | Potatoes |  |  |
|  |  | Sugar sweetened beverages^1^ |  |  |
|  |  | Sweets and desserts |  |  |
|  |  | Miscellaneous plant-based foods^2^ |  |  |
|  |  | *Plant-based Range* | *12 to 60* | |
| **Animal-based foods** |  | Animal added fats | Reverse score  (5 to 1 based on consumption quintiles) | Reverse score  (5 to 1 based on consumption quintiles) |
|  |  | Dairy foods |  |  |
|  |  | Eggs |  |  |
|  |  | Fish and seafood |  |  |
|  |  | Meat |  |  |
|  |  | Miscellaneous animal-based foods |  |  |
|  |  | *Animal-based Range* | *6 to 30* | |
| **Total Range** | **18 to 90** | | | |

Abbreviations: hPDI: healthy Plant-based Diet Index, uPDI: unhealthy Plant-based Diet Index

**List of modifications from the original study Satija et al.**
^1^ Clustering of “Fruit juices” and “Sugar-sweetened beverages” groups
^2^ Creation of the “Miscellaneous plant-based foods” group, including plant-based sugary or salty snacks

Supplemental 3: Food groups used to construct hPDI and uPDI scores

| **Categories** | **Groups** | **Description** |
| --- | --- | --- |
| Healthy plant-based food | Whole grains | All wholefoods including: Bread, Rusks, Low-sugar breakfast cereals, Pasta, Rice, Other cereals and Flours |
|  | Fruits | Fresh, low sugar stewed and canned fruit and dried fruit |
|  | Vegetables | Vegetables, starches, and tubers (other than potatoes) and vegetable soups |
|  | Nuts | Unsalted oil fruits and seeds, sesame puree, pine nuts |
|  | Legumes | Beans, flageolets, white, black and kidney beans, lentils, lupins, soybeans, split peas and chickpeas. Also include soy protein powder and tempeh |
|  | Vegetal oils | All oils without distinction in w3 and w6. |
|  | Tea Coffee | Tea, coffee, decaffeinated coffee |
| Unhealthy plant-based food | Refined Grains | Bread, Cereal wafers, Pizza dough, Sweetened breakfast cereals and cereal bars, Pasta (durum wheat, egg, fresh, with/without gluten, soy vermicelli), Rice (white, semi-complete), Semolina, Other cereals (Quinoa, bulgur, maize, sprouted seed, gluten (wheat protein), brewer's yeast), White flours (with/without gluten) |
|  | Potatoes | Chips, baked or mashed potatoes, potatoes or corn chips |
|  | Sugar sweetened beverages | Soft drinks, lemonade, sweetened soft drinks, syrup, iced tea, sweetened flavoured water and sweetened hot drinks. |
|  | Sweets and desserts | Cakes, Pastries, Sweets, Honey, Jam, Sugars, Chocolates, Ice cream, Spreads, Almond paste and Viennese pastries |
|  | Juice | Fruit juices from concentrate and nectars |
| Animal-based food | Animal fats | Sweet, light, semi-salted and salted butter. |
|  | Dairy products | Milk, Cheese, Yoghurt, Cottage cheese, Swiss cheese, Dairy desserts |
|  | Eggs | Eggs |
|  | Fishes and see-food | Fish, seafood and seafood delicatessen |
|  | Meat | Meat, Poultry, Pork and Poultry Hams, Offal and Charcuterie |
|  | Miscellaneous animal food | Snacks, savoury fast food and products |
